# Protein Conformational Changes Are Detected and Resolved Site Specifically by Second-Harmonic Generation

**DOI:** 10.1016/j.bpj.2015.07.016

**Published:** 2015-08-18

**Authors:** Ben Moree, Katelyn Connell, Richard B. Mortensen, C. Tony Liu, Stephen J. Benkovic, Joshua Salafsky

**Affiliations:** 1 Biodesy, Inc., South San Francisco, California; 2 Department of Chemistry, Pennsylvania State University, University Park, Pennsylvania

## Abstract

We present here a straightforward, broadly applicable technique for real-time detection and measurement of protein conformational changes in solution. This method is based on tethering proteins labeled with a second-harmonic generation (SHG) active dye to supported lipid bilayers. We demonstrate our method by measuring the conformational changes that occur upon ligand binding with three well-characterized proteins labeled at lysine residues: calmodulin (CaM), maltose-binding protein (MBP), and dihydrofolate reductase (DHFR). We also create a single-site cysteine mutant of DHFR engineered within the Met20 catalytic loop region and study the protein’s structural motion at this site. Using published x-ray crystal structures, we show that the changes in the SHG signals upon ligand binding are the result of structural motions that occur at the labeled sites between the apo and ligand-bound forms of the proteins, which are easily distinguished from each other. In addition, we demonstrate that different magnitudes of the SHG signal changes are due to different and specific ligand-induced conformational changes. Taken together, these data illustrate the potential of the SHG approach for detecting and measuring protein conformational changes for a wide range of biological applications.

## Introduction

The relationship between protein structure and function has long been recognized as fundamental for understanding biological mechanisms. Far from being static in structure, proteins are dynamic molecules that are capable of changing their shape, or conformation, in response to changes in their environment and upon ligand binding. By changing their conformation, proteins can carry out their functions and modulate the functions of other molecules. Conformational changes to the unliganded form of a protein provide the physical underpinning for allostery and signal transduction, and these changes are crucial for ubiquitous phenomena such as enzyme catalysis, protein-protein interactions, and motor protein movement (1–4). As our appreciation of proteins as dynamic, flexible molecules grows, so does the need for tools to probe the conformational landscape in real time and in physiological conditions.

**Figure 1:**
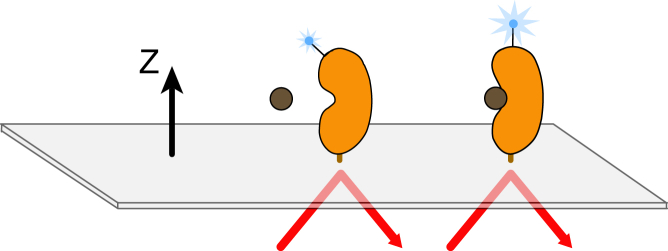
Schematic of the SHG experiment. Incident laser light strikes the surface and through total internal reflection creates an evanescent wave. Labeled protein is bound to the surface and the measured SHG signal magnitude depends on the average, net orientation of the dye label relative to the surface normal (*z* axis). A conformational change that alters the orientational distribution of the label in space or time results in a signal change.

Second-harmonic generation (SHG) is a nonlinear optical technique (5,6) in which two photons of equal energy are combined by a nonlinear material or molecule to generate one photon with twice the energy. This process is forbidden in a medium with centrosymmetry, but as symmetry is always broken at an interface, SHG is intrinsically surface selective. When second-harmonic-active molecules are immobilized at an interface and irradiated with a fundamental beam, typically pulsed at high peak intensity, the molecules radiate second-harmonic light in a coherent manner. Because SHG is highly sensitive to the orientation of second-harmonic-active molecules, the technique can be applied to study structure and conformational changes when a second-harmonic-active molecule is tethered to a surface. Although biological molecules are not usually second-harmonic active, they can be rendered so through the incorporation of a second-harmonic-active dye molecule (7). Once tethered to a surface, a labeled second-harmonic-active protein irradiated by a fundamental light source produces an SHG signal whose intensity depends sensitively on the tilt angle of the dye with respect to the surface normal (Fig. 1). When the protein undergoes a conformational change upon ligand binding, this causes a change in the time- and space-averaged orientation of the second-harmonic-active moiety, leading to a change in the intensity of light. This yields a real-time measurement that reports directly on a probe’s change in orientation at one or more labeled residues in a protein with high angular sensitivity. However, the potential utility of SHG for monitoring protein conformational changes has not been fully realized due to the lack of an easily accessible and biocompatible system.

To validate SHG as a broadly applicable biophysical technique for investigating protein structural motion, we examined three model proteins for studying ligand-induced conformational change: calmodulin (CaM), maltose-binding protein (MBP), and *Escherichia coli* dihydrofolate reductase (DHFR). CaM is a messenger protein that transduces calcium-generated signals by binding calcium ions (8). CaM is implicated in numerous biological processes, and the structural transition of CaM upon calcium ion binding has been extensively characterized by biochemical and biophysical techniques (9–13). Calcium-free CaM adopts an extended dumbbell structure with two similar lobes, each containing two calcium-ion-binding sites. Binding of calcium ions changes the relative orientation of the helices flanking the calcium-binding loops, exposing a hydrophobic surface region that serves as a binding site for target proteins. Upon binding of a target protein, the two lobes of CaM collapse at the central helix to fold around the target peptide.

MBP is a soluble, well-behaved protein for which the relationship among ligand binding, function, and conformational change has been extensively investigated by x-ray crystallography, NMR, and other biophysical techniques (14–21). MBP belongs to a class of molecules known as periplasmic-binding proteins, which are responsible for efficient uptake and catabolism of maltodextrins. Periplasmic-binding proteins share a two-domain structure linked by a flexible *β*-strand and are known to undergo large-scale motion from an open to a closed form upon maltose binding (22). The transition from the open to closed form of MBP has also been exploited as a platform for developing biosensors for specific target compounds (23,24).

DHFR is a popular enzyme model for studying the relationship between conformational change and catalysis (25–27). In cells, DHFR catalyzes the reduction of DHF to tetrahydrofolate, with NADPH as an electron donor (28). Tetrahydrofolate serves as cofactor in many reactions and is essential for purine and thymidylate biosynthesis, and thus cell growth, making DHFR a target for anticancer and antibacterial drugs (29). The conformation of the Met20 loop is particularly sensitive to ligand binding, and it adopts different shapes depending on which ligand is bound to the protein (27).

In the work presented here, we demonstrate a broadly applicable method based on SHG for studying protein conformational changes upon ligand binding in real time and under physiological conditions. We demonstrate that tethering of proteins to a biomimetic lipid membrane allows for facile capture of labeled protein molecules that retain their function, as shown by their ability to undergo well-characterized conformational changes. We demonstrate that different conformational changes induced by binding different ligands to the same labeled protein produce different responses by SHG. We also validate our SHG findings by identifying the labeled sites using mass spectrometry (MS) and correlating the motions we observe at these sites upon ligand binding with those observed in the x-ray crystal structures.

## Materials and Methods

### Preparation of lipids and supported lipid bilayers

The Ni-NTA bilayer surface used in this work is available from Biodesy (South San Francisco, CA). Preparation of the bilayer surface on glass was performed according to the manufacturer’s instructions. A characterization of protein binding to the bilayer surface is available in the Supporting Material (Figs. S1 and S2).

### Proteins and labeling

N-terminal poly-histidine-tagged MBP was purchased from AtGen (South Korea). N-terminal poly-histidine-tagged human CaM was obtained from EMD Millipore (Billerica, MA). N-terminus His-tag (8 His) *ec*DHFR variants were constructed using the Stratagene QuikChange site-directed mutagenesis kit and the wild-type (WT) *ec*DHFR template as described previously (30). For the His-tagged M20C variant, the two native cysteines (C85 and C152) in the WT enzyme were mutated to Ala and Ser, respectively, to generate a ΔCys *ec*DHFR as described previously (31,32). The choice of amino acid substitution (C85A/C152S) was shown to have no impact on enzymatic activity. Selective incorporation of cysteine was achieved through subsequent mutations using the following primer: 5′-GGC ATG GAA AAC GCC TGT CCA TGG AAC CTG-3′. Plasmid construction, protein expression, and purification of mutant DHFR proteins were performed according to a previously published protocol (30). The purified His-tag *E. coli* DHFR and its single-cysteine M20C derivative were found to have enzyme activity comparable to that of the WT enzyme. The His-tag WT enzyme, His-tag M20C, and non-His-tag WT *E. coli* DHFR exhibited hydride transfer rates of 205 ± 20 s^−1^, 180 ± 10 s^−1^, and 220 s^−1^ at pH 7 and 25°C (under standard kinetic conditions described previously (28)).

The second-harmonic-active dyes SHG1-SE (amine reactive, succinimidyl ester) and SHG2-maleimide (thiol reactive, maleimide) are available from Biodesy and were conjugated to each protein according to the manufacturer’s instructions.

### Sample preparation

Glass slides (Fisher) were cleaned in Piranha (30% H_2_O_2_, 70% H_2_SO_4_) at 100°C for 20 min. After cooling, the slides were washed five times with deionized water and dried with nitrogen gas. A custom 2-mm-thick silicone gasket template with adhesive backing (Arrowleaf Research, Bend, Oregon) was applied to the Piranha-cleaned slides. Each silicone gasket template defined 16 wells, each with a total volume of ∼14 *μ*L. The spacing and diameter of the wells were based on the standard 384-well-plate format. Small unilamellar vesicles (SUVs) were incubated with 1 mM of NiCl_2_ (Sigma, St. Louis, MO) for 30 min, diluted fivefold in buffer, and added to each well. After bilayer formation, the wells were washed with buffer to remove unbound SUVs and imaged by fluorescence microscopy to determine that the bilayer was fluid and uniform across the surface (33). Protein was then added to the wells at the desired concentration and allowed to incubate for a minimum of 1 h. Excess protein was removed by additional washes after incubation.

Each protein was screened to determine an optimized binding buffer for bilayer attachment and SHG signal production. For MBP, the optimized buffer was determined to be 20 mM Tris pH 8.0, 150 mM NaCl, 0.05% Tween-20, and 1 mM dithiothreitol. For CaM, the optimized buffer was 25 mM MOPS pH 7, 150 mM KCl, 2 mM MgCl_2_ (with or without 2 mM CaCl_2_). For DHFR, the optimized buffer was 50 mM sodium phosphate, pH 7.0, with 100 *μ*M NADPH (Sigma). For conformational-change experiments, 9 *μ*M MBP, 2 *μ*M CaM, or 4 *μ*M DHFR was incubated in each well for a minimum of 1 h. Stability experiments were carried out after overnight incubation in the wells at 4°C. For specificity experiments, each protein was incubated in 25 mM Tris pH 7.2 and 150 mM NaCl, with or without 300 mM imidazole. Excess protein and imidazole were removed by washing with 25 mM Tris pH 7.2, 150 mM NaCl before SHG data were acquired.

### SHG instrumentation and experiments

Our instrument comprises a mode-locked Ti:Sapphire system, which provides the fundamental beam necessary to generate the second-harmonic signal (high peak power). For these experiments, we used a Mira 900 Ti:Sapphire ultrafast oscillator (Coherent, Santa Clara, CA) pumped by a Millenia V DPSS laser (Spectra-Physics, Santa Clara, CA). The fundamental beam was passed through a half-wave plate to select p-polarization (used for all the experiments described here) and focused into a Dove prism at an angle of 69° (the critical angle for total internal reflection in our experiments) to a spot size of ∼100 *μ*m. The second-harmonic light, which emerges from the prism nearly collinear to the incident beam and parallel to the ground, was collected by a lens, separated from the fundamental beam using a dichroic mirror and wavelength filters, and directed into a PMT module with a built-in preamplifier for photon counting, using a 1-s integration time for each measurement (Hamamatsu, Bridgewater, NJ). No analyzer was used to select the polarization of the second-harmonic beam. A custom electronics board was used to digitize the signal, and the data were sent to a computer running customized control and data-collection software (Labview; National Instruments, Austin, TX).

For these experiments, the microscope slide with protein was coupled to a prism using BK7 index matching fluid (Cargille, Cedar Grove, NJ), and the prism itself was secured onto a 1D translation stage capable of 1 *μ*m randomly addressable precision (Renishaw, Parker-Hannifin, Rohnert Park, CA).

Ligand addition was carried out while the SHG signal was monitored in real time. Once the baseline signal was established, buffer was injected into the well as a control. After 5–10 s to assess how the buffer injection changed the signal, the compound of interest was injected into the well to the desired final concentration. The SHG signal was monitored for several minutes after injection.

### SHG quantification

To calculate the percent change in SHG intensity (%Δ_SH_), the second-harmonic intensity measured just before injection (I_t0_) was subtracted from the second-harmonic intensity at t_max_ (I_tmax_) and then divided by the initial second-harmonic intensity (I_t0_) according to the following equation:

(1) $$\%\Delta_{\mathrm{SH}}=\left(I_{\mathrm{tmax}}\text{-}I_{\text{t}0}\right)/I_{\text{t}0}.$$

In addition, all experiments included a control buffer injection that was used to determine the threshold for SHG intensity change and was calculated in a similar manner. All values in this work are reported as the mean ± SE of the percent change for each independent experiment.

### MS analysis

Liquid chromatography-tandem MS sample handling and protein identification were performed as a service by Martin Protean (Princeton, NJ).

## Results

### MBP and CaM proteins

**Figure 2:**
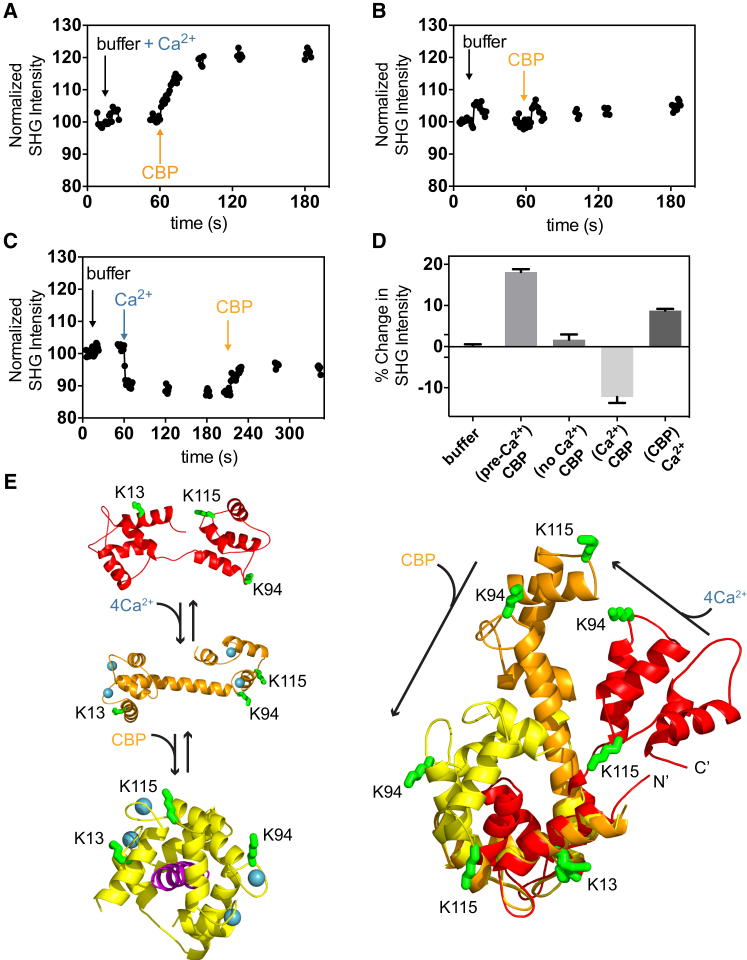
CaM. (*A–C*) Representative SHG time courses for addition of calcium and CBP to CaM. Arrows denote the time of addition of the indicated compounds. (*D*) Summary of the percent change in SHG signal observed after addition of calcium and CBP at a 2-min endpoint (*N* ≥ 3). (*E*) Crystal structures of free CaM (*red*; PDB ID: 1CFD), Ca-bound CaM (*orange*; PDB ID: 1CLL), and Ca/CBP-bound CaM (*yellow*; PDB ID: 1CDL) are shown with the labeled lysine residues identified by MS in green. Calcium ions are shown in blue and CBP is in purple.

To explore the ability of our SHG bilayer system to detect and measure the conformational changes of a protein, we began by testing the system on CaM and MBP, two proteins previously studied by SHG (34,35). First, we tethered labeled CaM to the supported lipid bilayer (SLB) surface and monitored the change in SHG intensity upon addition of calmodulin-binding peptide (CBP) in the presence or absence of calcium-containing buffers. As shown in Fig. 2 *A*, the addition of 2 *μ*M of CBP in the presence of a calcium-containing buffer resulted in a positive change in the SHG signal of 18.1% ± 0.6%, whereas buffer injection resulted in a signal change of 0.1% ± 0.4% (*N =* 9). When calcium was omitted from the buffer, addition of the peptide alone resulted in a 3.1% ± 1.6% change in SHG intensity (essentially no change; Fig. 2 *B*). As calcium-free CaM does not adopt a conformation capable of binding to peptide, these results confirm that the change observed upon peptide addition in the presence of calcium was due to the conformational change induced by peptide binding. Next, we evaluated the effect of adding calcium ions. When buffer containing 1 mM CaCl_2_ was injected into the system, a decrease in signal of −12.6% ± 1.4% resulted (Fig. 2 *C*). Peptide was then added directly to this sample 2 min after calcium addition, producing a positive signal change of 8.7% ± 0.4%. The difference in the magnitude of the observed signal change between the two experiments in which peptide was added is due to the difference in the CaCl_2_ concentrations and incubation times used. In the first experiment (Fig. 2 *A*), 2 mM of CaCl_2_ was incubated with CaM for 1 h before peptide addition, whereas in the second experiment, peptide was added 2 min after addition of 1 mM of CaCl_2_. The kinetics of calcium association and dissociation rates to and from the four binding sites on CaM were previously determined by rapid microfluidic mixing. Based on these measurements, and given that our measurements were performed in wells and kinetically limited by mass transport of the ligands to the surface-tethered protein, we would not expect the conditions of these two experiments to reach the same endpoint within 2 min. The magnitude of signal changes for each of these experiments at a 2-min endpoint is shown in Fig. 2 *D*. As the magnitude of the SHG signal is proportional to the net, average orientation of the dye label relative to the surface normal, the different magnitudes of the SHG signal change upon binding CBP and calcium to unbound CaM confirm that these ligands bind to specific and different conformations of the protein. SHG signal changes can occur in either a positive or negative direction relative to baseline depending on whether the net, average orientation of the probe moves closer to or farther away from the surface normal, respectively.

We next sought to determine which residues were modified with our amine-reactive dye. MS of labeled CaM revealed complete labeling at K115 and less than complete modification at K13 and K94. As the degree of labeling for CaM was measured to be 1.0 (dye/protein ratio) by UV-Vis spectroscopy, most of the signal was likely due to labeling at K115. As can be seen from the overlay of the crystal structures of the apo, calcium-bound, and CBP-bound CaM in Fig. 2 *E*, all three residues undergo large structural changes upon binding both calcium and CBP. Taken together, the data demonstrate that conformational changes associated with both calcium and CBP binding to CaM can be resolved using SHG. Moreover, the peptide- and calcium-induced conformational changes are clearly different in both magnitude and directionality, illustrating SHG’s ability to discriminate the different conformations the protein adopts upon binding different ligands.

**Figure 3:**
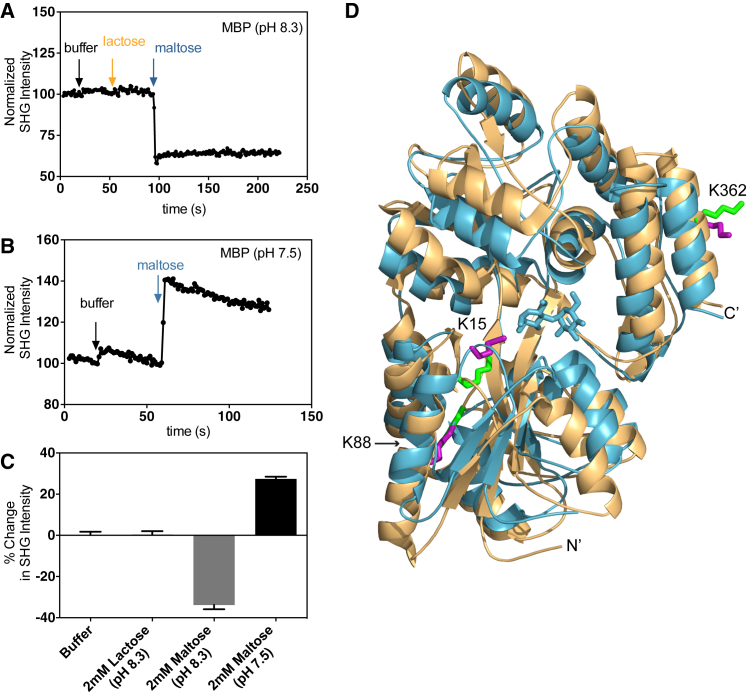
MBP. (*A*) Representative time course of an SHG signal during compound addition to MBP labeled at pH 8.3. The arrows denote the time of injection of buffer, lactose, and maltose. (*B*) Representative time course of an SHG signal during compound addition to MBP labeled at pH 7.5. The arrows denote the time of injection of buffer and maltose. (*C*) Summary of the percent change in SHG signal observed after addition of buffer, lactose, and maltose to MBP at a 2-min endpoint (*N* ≥ 3). (*D*) Overlay of the crystal structures of MBP with maltose (*blue*; PDB ID: 1ANF) and without maltose (*tan*; PDB ID: 1JW4) bound. Maltose is shown in blue. Modified lysine residues identified by MS are shown as sticks in green on the unbound structure and in purple on the bound structure. The labeled lysines are in different conformations in the two crystal structures. For this comparison, the structures were aligned at their N-terminal domains. The His-tag and thus the site of immobilization of the protein are at the N-terminus.

We also performed a similar set of experiments with MBP. First, we monitored the SHG intensity of MBP labeled at pH 8.3 upon addition of buffer, lactose, or maltose. As can be seen in the real-time trace of the SHG signal, the addition of 1 mM of maltose resulted in a rapid decrease of 33.8% ± 1.0%, whereas the addition of either buffer or 2 mM of lactose resulted in a negligible change of 0.12% ± 0.61% or 0.44% ± 0.79%, respectively (Fig. 3 *A*). The MBP system offers an excellent control in lactose, a stereoisomer of maltose that does not bind to MBP. Because the addition of both 1 mM of lactose and buffer alone resulted in negligible changes in SHG intensity, the change in SHG intensity upon maltose addition is specific to ligand-induced conformational changes upon binding.

The MS analysis of MBP revealed that it is heterogeneously labeled at K15, K88, K127, K239, K256, K297, K326, and K362, with residues K15, K88, and K362 representing ∼90% of the total population of modified peptides. The degree of labeling for MBP was 1.3, confirming that more than one residue was labeled. We aligned the crystal structures of the apo and maltose-bound MBP and compared the positions of the labeled residues in these two structures. As can be seen in Fig. 3 *D*, the side chains of the labeled residues show significant orientational differences, providing further validation that the observed change in SHG intensity is the result of ligand-induced binding of maltose to MBP.

As MBP demonstrated a high degree of lysine modifications, we modified our labeling conditions to explore whether we could target specific lysines for labeling. Labeling MBP at pH 7.5 rather than pH 8.3 significantly increased the overall abundance of the K15 modification, with a corresponding decrease in the modification at K88: 70% of the peptides detected by MS showed labeling at K15 rather than K88. The converse trend, a preference for K88 over K15, was observed when the experimental conditions were changed to pH 8.3. The degree of labeling of the conjugate labeled at pH 7.5 was 1.3, which suggests that the protein was primarily labeled at K15. This example shows that by varying the conditions of the conjugation reaction, one can bias the distribution of labeled lysines, most likely by exploiting the differences in each residue’s microenvironment and p*K*_a_. Based on inspection of the crystal structures, we hypothesized that when MBP is primarily labeled at K15 rather than K88, this could alter the directionality of SHG signal changes upon ligand addition relative to the reverse case, since K15 and K88 appear to rotate toward and away from the surface normal, respectively, assuming the protein is oriented with its N-terminus facing the bilayer. As can be seen in Fig. 3 *B*, addition of 1 mM of maltose to pH 7.5-labeled MBP resulted in an increase in SHG intensity of 27.4% ± 0.61%. Consistent with previous results, the addition of lactose and buffer alone had no effect. These results are summarized in Fig. 3 *C*.

### DHFR protein

**Figure 4:**
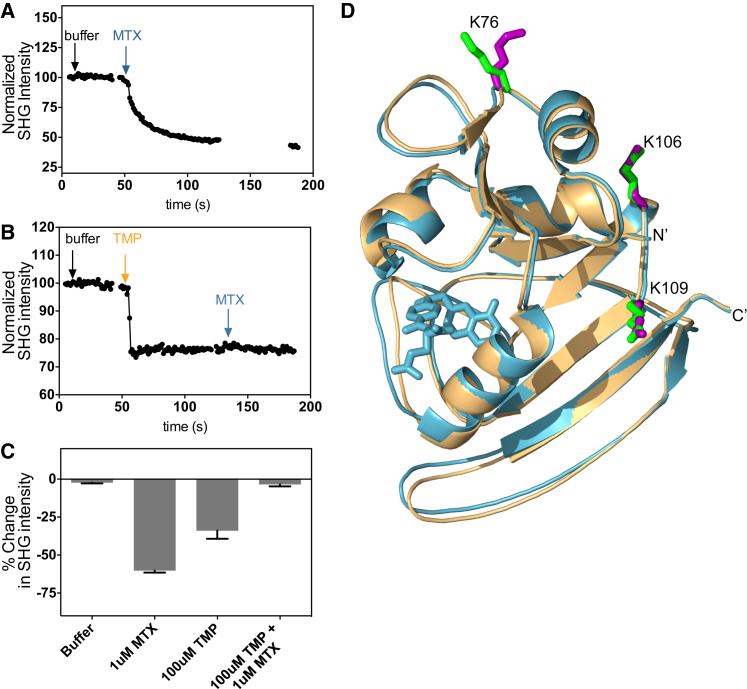
Amine-labeled DHFR. (*A*) Representative kinetic trace for MTX addition to lysine-labeled DHFR. Arrows denote the time of addition of buffer and MTX. (*B*) Representative kinetic trace for a TMP-MTX competition experiment with lysine-labeled DHFR. Arrows denote the time of addition of buffer, TMP, and MTX. (*C*) Summary of percent change of SHG signal observed after addition of buffer, MTX, and TMP to lysine-labeled DHFR at a 2-min endpoint (*N* ≥ 4). (*D*) Crystal structures of DHFR holoenzyme with (*blue*; PDB ID: 1RB3) and without (*tan*; PDB ID: 1RX1) MTX bound. Labeled residues identified by MS are shown as sticks in green for DHFR without MTX, and in purple for DHFR bound to MTX. MTX is represented in blue. NADPH (100 *μ*M) was present in all experiments.

As a final demonstration of the technique, we studied *E. coli* DHFR, a protein previously uncharacterized by SHG. In particular, we focused on the protein’s response to two important pharmaceutical inhibitors: methotrexate (MTX) and trimethoprim (TMP) (29). For these studies, we used two different approaches to label the protein: native lysine residues and an engineered, unique cysteine at residue 20. As shown in Fig. 4 *A*, the addition of 1 *μ*M of MTX to the amine-labeled DHFR resulted in a rapid decrease in the SHG signal of −60.2% ± 1.26%, compared with −2.27% ± 0.48% (*N =* 8) for the buffer control. We next explored whether TMP addition by itself would result in a conformational change in DHFR. As can be seen in Fig. 4 *B*, the addition of 100 *μ*M of TMP resulted in a decrease in SHG intensity of −33.9% ± 5.31%. It is known that MTX and TMP bind competitively to the same site on DHFR. Subsequent addition of 1 *μ*M of MTX after 100 *μ*M of TMP resulted in a decrease in the SHG signal of −3.48% ± 1.31%, a negligible change. If the TMP-induced SHG signal change were due to loss of protein from the surface rather than conformational change, addition of MTX after TMP should have resulted in a further signal change, since ample baseline signal (and thus protein) remained after the TMP addition. However, MTX’s response was completely blocked after the TMP addition, demonstrating that the observed signal changes were due to a conformational change rather than loss of protein from the surface. The signal changes we observed for these experiments are summarized in Fig. 4 *C*. MS analysis revealed that three residues, K76, K106, and K109, were modified by the amine-reactive SHG dye, with the modification at K76 being nearly complete. The degree of labeling of the amine-labeled DHFR conjugate was 1.1, suggesting that labeled K76 was the primary contributor to the observed signal. As can be seen in an overlay of the x-ray structures of the apo and MTX-bound DHFR holoenzyme (Fig. 4 *D*), although there is relatively little motion at sites K106 and K109, there is a significant reorientation at site K76, corroborating our SHG measurements and the MS analysis.

**Figure 5:**
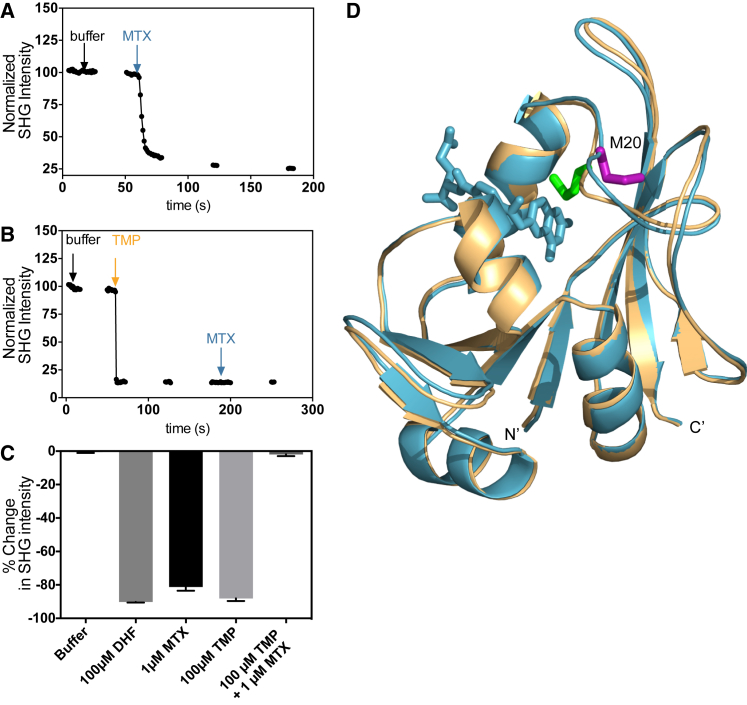
Cysteine-labeled DHFR. (*A*) Representative time course for MTX addition to cysteine-labeled DHFR. (*B*) Representative kinetic trace for a TMP-MTX competition experiment with cysteine-labeled DHFR. Arrows denote the time of addition of buffer, TMP, and MTX. (*C*) Summary of the percent change of SHG signal observed after addition of buffer, DHF, MTX, and TMP to cysteine-labeled DHFR at a 2-min endpoint (*N* ≥ 3). (*D*) Crystal structures of DHFR holoenzyme with MTX (*blue*; PDB ID: 1RB3) and without MTX (*tan*; PDB ID: 1RX1) bound. Residue M20 is shown in purple and green sticks for the bound and unbound forms, respectively. MTX is represented in blue. NADPH (100 *μ*M) was present in all experiments.

We also performed the same series of experiments on DHFR labeled specifically at a single-site engineered cysteine (M20C), which participates in the key catalytic steps of the enzyme. Complete labeling of the cysteine residue was confirmed by MS and the degree of labeling was 1.0, indicating that the labeling was site specific. The ΔCys M20C construct maintained the WT enzymatic activity described in the Materials and Methods section. As can be seen in Fig. 5 *A*, the trends are similar to those observed with amine-labeled DHFR, although the magnitude of the observed change is different. Addition of MTX to cysteine-labeled DHFR on the bilayer resulted in a large change in SHG intensity of −81.03% ± 2.4%, compared with −0.6% ± 0.4% for buffer alone. The addition of TMP alone produced a similar decrease in SHG intensity of −88.05% ± 1.5%, and, as with amine-labeled DHFR, addition of 1 *μ*M MTX in the presence of TMP resulted in an insignificant change in SHG intensity of −1.9% ± 1.0% (Fig. 5 *B*). In addition to the antifolate compounds, we also tested the natural ligand DHF on the M20C-labeled DHFR. We found that the addition of 100 *μ*M of DHF resulted in a signal change of −90.2% ± 0.4%. These results are summarized in Fig. 5 *C*. In addition, we performed a dose-response experiment using TMP, and determined its EC_50_ for binding to DHFR as logEC_50_ of −8.05 ± 0.07 (EC_50_ = 8.9 nM) (Fig. S3), which is in excellent agreement with published values (31). This experiment demonstrates that SHG has the sensitivity to detect binders with even nanomolar affinity. Taken together, these results suggest that the ternary complexes of DHFR/NADPH bound to DHF, MTX, and TMP are similar, in agreement with the published crystallographic structures of inhibitor-bound DHFR (36). Moreover, the results suggest that the potency of the antifolate inhibitors MTX and TMP is due to their ability to mimic the conformation induced by binding the natural substrate.

## Discussion

In the work described here, we developed a broadly applicable and sensitive approach that uses SHG to detect and resolve ligand-induced protein conformational changes. We demonstrated the method using three different proteins (CaM, MBP, and *E. coli* DHFR). This approach is based on using SHG-labeled proteins tethered to an SLB membrane. Although most proteins are not intrinsically second-harmonic active, they can easily be made so by standard amine- and thiol-reactive chemistries. We identified the labeled sites by MS, which in combination with available x-ray crystal structures allowed us to inspect and correlate the structural motions we observed by SHG with the changes in orientation observed at the modified residues in both the apo and bound structures for all three proteins. Importantly, we found that the labeling site(s) determined by MS for both the amine- and cysteine-labeled conjugates for the three proteins studied here was remarkably consistent between preparations when the reaction conditions of the conjugation, including the pH, probe/protein molar ratios, and concentrations of the reactants, were held constant.

In addition to providing direct evidence that the changes in the SHG signal upon ligand binding result from motion at specific labeled residues, the MS analysis also provided indirect support for the orientation of the protein on the bilayer. Because the intensity of the SHG signal is directly dependent on the net, average orientation, we can infer the motion at the predominantly labeled residues using the direction of the SHG signal change. For example, because the signal change in DHFR upon MTX addition decreases, we would expect the K76 side chain to move away from the surface normal. The protein is tethered to the surface through the N-terminal poly-histidine tag, and a large rotation of the K76 side chain away from the normal upon MTX addition is easily seen by inspection of the crystal structures. Only small motions are observed for the other modified residues, K106 and K109, suggesting that K76 contributes the majority of the motion we observe in amine-labeled DHFR samples.

We also tested our ability to target specific label sites by changing the pH of the conjugation reaction. At lower pH, the reduction of the K88 modification and subsequent enrichment of the K15 modification under these conditions resulted in the SHG response of MBP binding to maltose switching directionality, from a decrease in intensity relative to baseline for protein labeled at pH 8.3 to an increase in intensity for protein labeled at pH 7.5. Since the MS analysis revealed no other differences between the conjugates labeled at these two pH values, this change in signal directionality is most likely due to the difference in the direction of movement between residues K15 and K88. The motion of the K88 residue upon MBP binding should cause a decrease in SHG signal intensity with the protein tethered to the surface through the N-terminal poly-histidine tag, consistent with our observations. In contrast, with labeling predominantly at K15, we would expect an increase in SHG signal intensity, which would also be consistent with the SHG results.

In addition to SHG’s sensitivity, the technique offers a number of advantages over traditional methods for probing conformational changes, one of which is the relative ease of performing experiments, as no a priori structural knowledge or mutagenesis is required. The only requirements for sample preparation are the incorporation of a poly-histidine tag and the labeling of the target protein to make it second-harmonic active. Multiple approaches can be used to label proteins as we have demonstrated here, including labeling of lysines through amine-reactive chemistry, site-specific labeling of native or engineered cysteines through thiol-reactive chemistry, and preferentially favoring specific residues by changing the conjugation conditions.

Moreover, there are no restrictions on the size or type of protein that can be studied by SHG; high-molecular-weight proteins, protein complexes, and intrinsically disordered proteins can all be studied. Likewise, the technique is amenable to a wide variety of experimental conditions and ligands, ranging from chemical fragments to small molecules and larger proteins for probing small-molecule- and protein-protein interactions, because the technique does not depend on mass accumulation to produce a signal. The modest protein requirement for SHG measurements should allow large-scale, structure-based screens that are not currently possible with other biophysical methods, which generally require much higher amounts of protein. In addition, the relatively low protein concentration requirement indicates that the technique lends itself well to the study of proteins that are not soluble or are prone to aggregation at the higher concentrations required for techniques such as NMR and x-ray crystallography.

Furthermore, the technique is well suited for structure-activity relationship (SAR) experiments. The SHG signals are very reproducible, and the differences in signal produced by two different ligands—and thus conformations—can be easily distinguished. In particular, the ability to resolve different ligands by the distinct and specific conformational changes they produce, and to correlate these changes with activity measurements (functional, pharmacological, etc.) offers a direct way to conduct SAR experiments to understand the connections between ligand structure, protein conformation, and function. Here, we show that *E. coli* DHFR produced different SHG responses when the enzyme was bound to two different pharmaceutical inhibitors, MTX and TMP. Knowing that the inhibition constant (Ki) of MTX is ∼100 times greater than that of TMP, one might be able to establish an SAR by evaluating the Ki value against the magnitude of the SHG signal change and thus the different conformations produced upon binding of different ligands. In addition, one could establish other SAR correlations by correlating in vivo data with the conformational changes produced upon binding of different ligands. In general, once an SAR is established, the SHG platform offers a more convenient and efficient way to screen for potential drug candidates compared with current activity and kinetic-based assays, by classifying the conformational changes of compounds into groups that produce different functional outcomes.

## Conclusions

In summary, our data demonstrate that the method presented here sensitively detects ligand-induced conformational changes that range in magnitude from the relatively small rotation of an amino acid side chain to the global motion of protein domains. Enabled by a biomimetic SLB as a two-dimensional platform for tethering and orienting proteins, the highly sensitive and generally applicable SHG structural technique described here can be used for many facets of research, such as probing the functional implications of protein structural rearrangements due to ligand, drug, or protein-protein interactions, as well as mutational screening.

The instantaneous, real-time nature of SHG offers investigators the opportunity to obtain measurements on even faster timescales than are currently possible to study how the kinetics and dynamics of conformational changes engender protein function. We expect that such measurements will be a focus of future studies. Finally, this work lays the foundation for future polarization-dependent studies (5,6,34,37–41) in which the probe orientational distribution at one or more sites, and thus the conformational landscape of the protein, can be mapped.

## Author Contributions

B.M., R.M., S.B., and J.S. designed the experiments. B.M., K.C., R.M., and T.L. performed experiments and analyzed data. B.M., K.C., T.L., S.B., and J.S. wrote the manuscript. S.B. and J.S. supervised the work.

## Supporting Citations

References (42–48) appear in the Supporting Material.

## Supporting Material

Document S1. Supporting Materials and Methods, Supporting Results, and three figures

Document S2. Article plus Supporting Material
