# Supplementary material for: Protein Conformational Changes Are Detected and Resolved Site Specifically by Second-Harmonic Generation
Source: Biophys J. 2015 Aug 18;109(4):806–15. doi: 10.1016/j.bpj.2015.07.016 (PMC4547196; doi:10.1016/j.bpj.2015.07.016)
Supplement: Document S1. Supporting Materials and Methods, Supporting Results, and three figures [file mmc1.pdf]

Biophysical Journal

Supporting Material

Protein Conformational Changes Are Detected and Resolved Site Specifically by Second-Harmonic Generation

Ben Moree[1], Katelyn Connell,[1] Richard B. Mortensen,[1] C. Tony Liu,[2] Stephen J. Benkovic,[2] and Joshua Salafsky[1,]*

[1]Biodesy, Inc., South San Francisco, California; and [2]Department of Chemistry, Pennsylvania State University, University Park, Pennsylvania

Supporting Material

Supporting Results.

Characterization of protein attachment to the SLB. Both CaM and MBP have previously been examined for conformational transitions upon ligand binding using SHG (1, 2). However, in those experiments protein immobilization on the surface was accomplished using aldehyde-derivatized glass slides to covalently couple the protein via amine-containing residues to the surface. While tethering the protein directly to the slide achieves the noncentrosymmetric distribution required for SHG, this method of attachment is far from ideal as proteins immobilized in this manner have been shown to unfold or lose secondary structure (3, 4). Because the direct attachment of protein to derivatized glass surfaces very often does not result in tethered and functional protein, we developed an SLB interface as an attachment platform for SHG. SLBs are biomimetic and they have been used extensively in many biochemical and cell biology experiments with minimal impact on protein structure and function (5-7).

We began by characterizing the attachment of the proteins to our SLB surface. The SLB surface we employed has integrated immobilized metal affinity (Ni-NTA) chemistry to specifically tether the proteins to the bilayer in an oriented manner via a poly-histidine tag at the N- or C-terminus. To test the specificity of this interaction, each protein was incubated on the bilayer in the presence or absence of imidazole, a competitive inhibitor of His-tagged protein binding. As can be seen in Fig. S1-A, incubation of the second-harmonic-functionalized proteins in the absence of imidazole results in a large increase in the SHG signal compared to the controls (addition of the unlabeled proteins or just the bilayer alone). When labeled protein is incubated in the presence of imidazole, SHG signal levels are reduced to less than 10% of the original signal or to background levels, i.e., to those of bilayer alone. Taken together, these results show that the observed SHG signal arises specifically from the dye-labeled protein bound via the His-tag-Ni-NTA linkage.

Next, we investigated the stability of the protein tethered to the SLB surface. Although the poly-histidine tag allows the protein to be captured and oriented in a specific manner on the SLB surface, the affinity of the 6x-His tag for Ni-NTA is only on the order of 100 nanomolar to the low micromolar range (0.1-1 μM) (8). Given this affinity range, we wanted to characterize the kinetics of protein loss from the SLB surface to the bulk solution after washout of the unbound protein fraction, as loss of protein due to unbinding from the surface could impact the measured change in SHG intensity over the time course of our experiments. Protein was incubated overnight at 4 °C, as it has been reported that the stability of poly-histidine tagged proteins on bilayers is greatly increased by longer incubation periods (9). Following washout of unbound protein, the SHG signal for each protein was monitored over the course of about one hour. Fig. S1-B shows that after washout the SHG signal is nearly constant over one hour, with losses of 3% observed for DHFR and 16% for MBP, demonstrating minimal dissociation of protein from the surface over this time frame. The stability of the protein attachment to the bilayer provides confidence that signal changes observed during our experiments, which occur

over seconds to a few minutes, are the result of conformational change and not protein dissociation from the surface. The stability of our system also enables the monitoring of relatively slow processes on the order of tens of minutes.

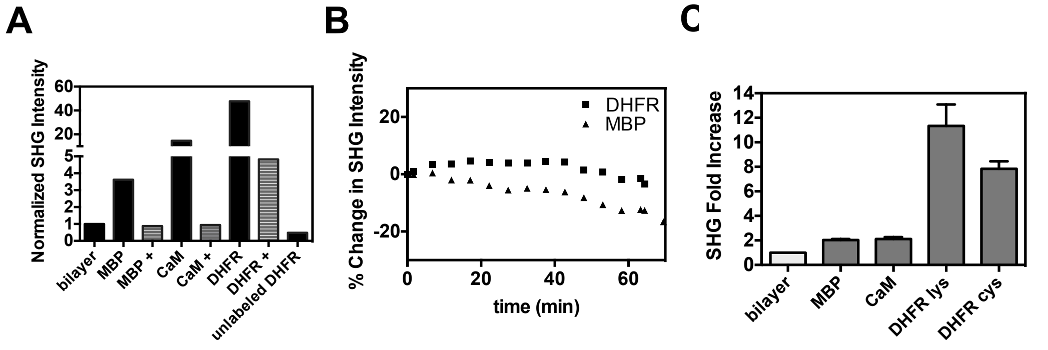

Fig. S1: Characterization of protein immobilization on the bilayer surface (A) Bar graph showing the intensity of the SHG signal for the model proteins, with and without imidazole, and for unlabeled protein compared to bilayer. The + denotes the presence of 300 mM imidazole. (B) SHG signal was monitored as a function of time after washout of excess protein from the bilayer surface. (C) Bar graph showing the intensity of the SHG signal from incubation with 100 ng of each of the model proteins on the bilayer (*N*=3).

To further characterize our system, we explored the amount of protein required for producing reliable SHG signals on the bilayer surface. In these experiments, 100 ng of each of the proteins was incubated on the bilayer for one hour, followed by buffer washout of excess protein. For each protein, signal of at least two-fold over background is observed (Fig. S1-C). Finally, we measured the amount of the lysine-labeled MBP and DHFR proteins tethered to the membrane after washout with buffer. To do so, we tethered the proteins to the SLB, washed out the unbound protein with buffer, and then solubilized both the protein and the bilayer with detergent (Fig. S2). The SHG label is also fluorescent and its signal intensity was measured by fluorometry and compared to a standard curve of the same labeled proteins. At the incubation concentrations in the experiments described here, lysine-labeled MBP (pH 8.3 conjugation) and DHFR show tethered surface densities of $4.9 \pm 0.7 \times 10^{12}$ molecules/cm^2 and $2.8 \pm 0.1 \times 10^{12}$ molecules/cm^2, respectively. This would correspond to approximately 14% of the $\sim 3 \times 10^{13}$ molecules/cm^2 maximum for both proteins given their physical dimensions and theoretical close-packed density. Taken together, the data characterizing our SLB surface demonstrate that SHG-labeled poly-histidine-tagged proteins are specifically and stably attached to the bilayer surface via metal chelation, that an SHG signal over background can be generated from bulk incubation with as little as 100 ng of labeled protein, and that the measured SHG signals are generated by less than a monolayer of protein molecules.

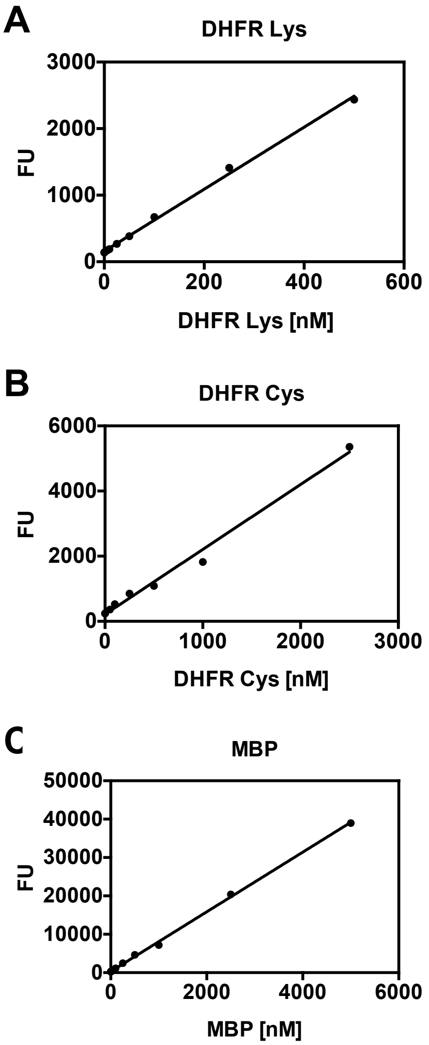

Fig. S2. Standard curves of the fluorescence from labeled proteins. Standard curves of cysteine-labeled DHFR (N=5, R^2=0.99) (A), lysine-labeled DHFR (N=5, R^2=0.98) (B) and MBP (N=5, R^2=0.99) (C). Tethered and labeled protein attached to the Ni-NTA bilayer was removed from the glass surfaces by serial detergent washes. Cysteine-labeled DHFR, lysine-labeled DHFR and MBP were all determined to be tethered at surface densities of order 10^{12} molecules per cm^2.

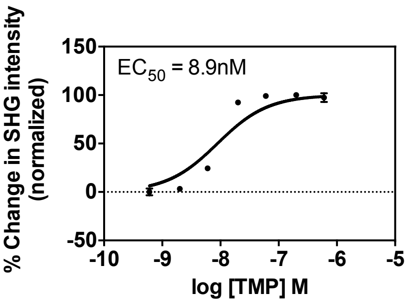

Fig. S3. TMP dose-response curve. Serial dilutions of TMP were injected into wells containing 1 µM maleimide-labeled DHFR + 100 µM NADPH. The TMP concentrations tested were 0.6 nM, 2 nM, 6 nM, 20 nM, 60 nM, 200 nM and 600 nM. Sixteen injections were performed at each TMP concentration to ensure that the ligand was at saturation for all concentrations and to overcome the mass transport limitation of the compound binding to the surface-tethered protein. The total percent change was recorded after the final injection. The data were normalized so that the values for the lowest concentration and the highest concentration were set to 0 and 100%, respectively. The dose response was then calculated by plotting the concentration (on the logarithmic scale) versus the percent change using a nonlinear regression fit for log (agonist) vs. normalized response in Prism (GraphPad Software, La Jolla, CA, USA). EC_{50} values were calculated empirically using the software. N = 4. Error bars = SD.

Supporting Methods

Quantification of the surface density of protein molecules. Four wells each of MBP and DHFR were incubated in wells containing supported lipid bilayer at 4 µM in their respective buffers. After one hour, wells were washed 5 x 20 µL with the appropriate buffer, and SHG signal was measured to confirm binding. A 10-µL aliquot of PBS/LDAO was used to wash the first well and this was carried forward into three other wells so that protein was collected from a total of four replicate wells. Five more 10-µL aliquots of PBS/LDAO were used to wash out the wells in the same manner so that in total 6 x 10-µL aliquots were used to wash out the four wells. Four wells were used to increase the fluorescence signal-to-noise and to average over separate wells. The wash was collected and monitored for second-harmonic-active dye fluorescence on a Horiba Jobin Yvon Fluorolog F-1000 fluorometer in a 384-well plate. Second-harmonic-active dye was excited at 415 nm and emission was collected from 520 to 620 nm. Standard curves for each labeled protein were generated to calculate the concentration of protein in the samples from the slides, which was used to determine the number of molecules tethered to the surface in one well.

Supporting References

1. Salafsky, J. S. 2006. Detection of protein conformational change by optical second-harmonic generation. The Journal of chemical physics 125:074701.
2. Salafsky, J. S. 2003. Second-harmonic generation as a probe of conformational change in molecules. Chemical Physics Letters 381:705-709.
3. Menaa, B., M. Herrero, V. Rives, M. Lavrenko, and D. K. Eggers. 2008. Favourable influence of hydrophobic surfaces on protein structure in porous organically-modified silica glasses. Biomaterials 29:2710-2718.
4. Menaa, B., C. Torres, M. Herrero, V. Rives, A. R. Gilbert, and D. K. Eggers. 2008. Protein adsorption onto organically modified silica glass leads to a different structure than sol-gel encapsulation. Biophysical journal 95:L51-53.
5. Manz, B. N., B. L. Jackson, R. S. Petit, M. L. Dustin, and J. Groves. 2011. T-cell triggering thresholds are modulated by the number of antigen within individual T-cell receptor clusters. Proceedings of the National Academy of Sciences 108:9089-9094.
6. Dustin, M. L., and J. T. Groves. 2012. Receptor Signaling Clusters in the Immune Synapse. Annual Review of Biophysics 41:543-556.
7. Alaimo, A., A. Alberdi, C. Gomis-Perez, J. Fernandez-Orth, G. Bernardo-Seisdedos, C. Malo, O. Millet, P. Areso, and A. Villarroel. 2014. Pivoting between calmodulin lobes triggered by calcium in the Kv7.2/calmodulin complex. PloS one 9:e86711.
8. Block, H., B. Maertens, A. Spriestersbach, N. Brinker, J. Kubicek, R. Fabis, J. Labahn, and F. Schafer. 2009. Immobilized-metal affinity chromatography (IMAC): a review. Methods in enzymology 463:439-473.
9. Nye, J. A., and J. T. Groves. 2008. Kinetic control of histidine-tagged protein surface density on supported lipid bilayers. Langmuir : the ACS journal of surfaces and colloids 24:4145-4149.
